# Novel germline variants in *KMT2C* in Chinese patients with Kleefstra syndrome-2

**DOI:** 10.3389/fneur.2024.1340458

**Published:** 2024-01-31

**Authors:** Qi Yang, Qiang Zhang, Sheng Yi, Shujie Zhang, Shang Yi, Xunzhao Zhou, Zailong Qin, Biyan Chen, Jingsi Luo

**Affiliations:** ^1^Guangxi Key Laboratory of Birth Defects Research and Prevention, Guangxi Key Laboratory of Reproductive Health and Birth Defects Prevention, Maternal and Child Health Hospital of Guangxi Zhuang Autonomous Region, Nanning, China; ^2^Department of Genetic and Metabolic Central Laboratory, Maternal and Child Health Hospital of Guangxi Zhuang Autonomous Region, Nanning, China; ^3^Guangxi Clinical Research Center for Pediatric Diseases, Maternal and Child Health Hospital of Guangxi Zhuang Autonomous Region, Nanning, China

**Keywords:** Kleefstra syndrome-2, KMT2C, novel variants, whole-exome sequencing, genotype-phenotype correlation

## Abstract

Kleefstra syndrome (KLEFS) refers to a rare inherited neurodevelopmental disorder characterized by intellectual disability (ID), language and motor delays, behavioral abnormalities, abnormal facial appearance, and other variable clinical features. KLEFS is subdivided into two subtypes: Kleefstra syndrome-1 (KLEFS1, OMIM: 610253), caused by a heterozygous microdeletion encompassing the *Euchromatic Histone Lysine Methyltransferase 1* (*EHMT1*) *gene* on chromosome 9q34.3 or pathogenic variants in *the EHMT1* gene, and Kleefstra syndrome-2 (KLEFS2, OMIM: 617768), caused by pathogenic variants in the *KMT2C* gene. More than 100 cases of KLEFS1 have been reported with pathogenic variants in the *EHMT1* gene. However, only 13 patients with KLEFS2 have been reported to date. In the present study, five unrelated Chinese patients were diagnosed with KLEFS2 caused by *KMT2C* variants through whole-exome sequencing (WES). We identified five different variants of the *KMT2C* gene in these patients: c.9166C>T (p.Gln3056^*^), c.9232_9247delCAGCGATCAGAACCGT (p.Gln3078fs^*^13), c.5068dupA (p.Arg1690fs^*^10), c.10815_10819delAAGAA (p.Lys3605fs^*^7), and c.6911_6912insA (p.Met2304fs^*^8). All five patients had a clinical profile similar to that of patients with KLEFS2. To analyze the correlation between the genotype and phenotype of KLEFS2, we examined 18 variants and their associated phenotypes in 18 patients with KLEFS2. Patients carrying *KMT2C* variants presented with a wide range of phenotypic defects and an extremely variable phenotype. We concluded that the core phenotypes associated with *KMT2C* variants were intellectual disability, facial dysmorphisms, language and motor delays, behavioral abnormalities, hypotonia, short stature, and weight loss. Additionally, sex may be one factor influencing the outcome. Our findings expand the phenotypic and genetic spectrum of KLEFS2 and help to clarify the genotype–phenotype correlation.

## Introduction

Kleefstra syndrome (KLEFS) is a rare inherited neurodevelopmental disorder (NDD) characterized by neuropsychiatric anomalies, developmental delay (DD), mild-to-severe intellectual disability (ID), childhood hypotonia, speech delay, and craniofacial abnormalities (1, 2). Globally, the prevalence stands between 1:25,000 and 1:35,000 individuals. KLEFS is primarily caused by a heterozygous microdeletion encompassing the *Euchromatin Histone Methyltransferase 1* (*EHMT1*) gene on chromosome 9q34.3 or a pathogenic variant in the *EHMT1* gene (3, 4). Additionally, pathogenic variants in KMT2C have been found in patients with a clinical diagnosis of Kleefstra syndrome-2. *KMT2C (KMT2C;* MIM 606833, NM_170606) is located on chromosome 7q36 and consists of 59 exons, encoding lysine-*N*-methyltransferase 2C, which catalyzes histone 3 lysine 4 (H3K4) methylation and epigenetically regulates gene transcription through chromatin modifications (5). Dysregulated H3K4 methylation can cause ID, epilepsy, autism spectrum disorder (ASD), schizophrenia, and other neurodevelopmental and neuropsychiatric disorders (6, 7). Recently, novel or de *novo* pathogenic variants in the *KMT2C* gene have been reported to cause a variety of developmental abnormalities, including ID, ASD, schizophrenia, and non-syndromic primary tooth eruption failure (8–13). To date, only 13 cases of KLEFS2 have been reported in the literature (8, 14–18). Currently, there is no clear association between the clinical phenotype and variant sites. Thus, additional reports would help us better understand the phenotype spectrum of this disease and explore the relationship between genotypes and phenotypes.

In this study, we present five additional patients diagnosed with Kleefstra syndrome-2 by whole-exome sequencing with a highly heterogeneous phenotype, including mild-to-moderate ID, facial dysmorphism, language and motor delays, short stature, weight loss, microcephaly, and behavioral abnormalities. Molecular analyses identified five novel *KMT2C* variants. Additionally, we have reviewed the literature to summarize the genotypes, phenotypes, and clinical features of KLEFS2.

## Materials and methods

### Subjects

For this study, five unrelated patients with confirmed *KMT2C* variants, who were diagnosed with neuropsychiatric anomalies, intellectual disability, global developmental delay, and craniofacial abnormalities, were recruited from the Maternal and Child Health Hospital of Guangxi Zhuang Autonomous Region (Table 1).

**Table 1:** Clinical features of the patients with KMT2C mutations.

**Patients clinical data**	**Patient 1**	**Patient 2**	**Patient 3**	**Patient 4**	**Patient 5**
Variants in KMT2C (NM_170606.2)	c.9166C>T (p.Gln3056 ^*^)	c.9232_9247delCA GCGATCAGAACCGT (p.Gln3078fs^*^13)	c.5068dupA (p.Arg1690fs^*^10)	c.10815_10819delA AGAA (p.Lys3605fs^*^7)	c.6911_6912insA (p.Met2304fs^*^8)
Inherit	*de novo*	*de novo*	*de novo*	*de novo*	Maternal
Gender	Male	Male	Female	Male	Male
Age at last examination	4 years	1 years	9 years 5months	14 years	8 years
Height (SD)	−3	−2	−2	−4.1	−1.2
Weight (SD)	−4.2	−2	−3	0	−2
OFC (SD)	0	−1.9	0	−0.4	−0.5
Failure to thrive	+	+	+	–	+
Walking	25 months	Can't walk at last examination	15 month	Normal	18 months
Speech impairment	Delayed speech	Delayed speech	Delayed speech	Delayed speech	Delayed speech
Intellectual disability	Moderate	Moderate	Mild	Mild	Moderate
Facial dysmorphism	NA	+	+	–	+
Muscular hypertonia	+	+	+	–	+
Behavioral abnormalities	Autism	–	ADHD	Sleeping disorder	Autism
Brain radiologic features	Normal	Normal	Normal	Normal	Normal
Seizures	–	–	–	–	–
Other	–	–	–	–	–

The following abbreviations are used: N.r., not reported; SD, standard deviation; GH, growth hormone; ADHD, attention-deficit-hyperactivity disorder; OFC, occipitofrontal circumference.

### Whole-exome sequencing and sanger sequencing

**Figure 1:**
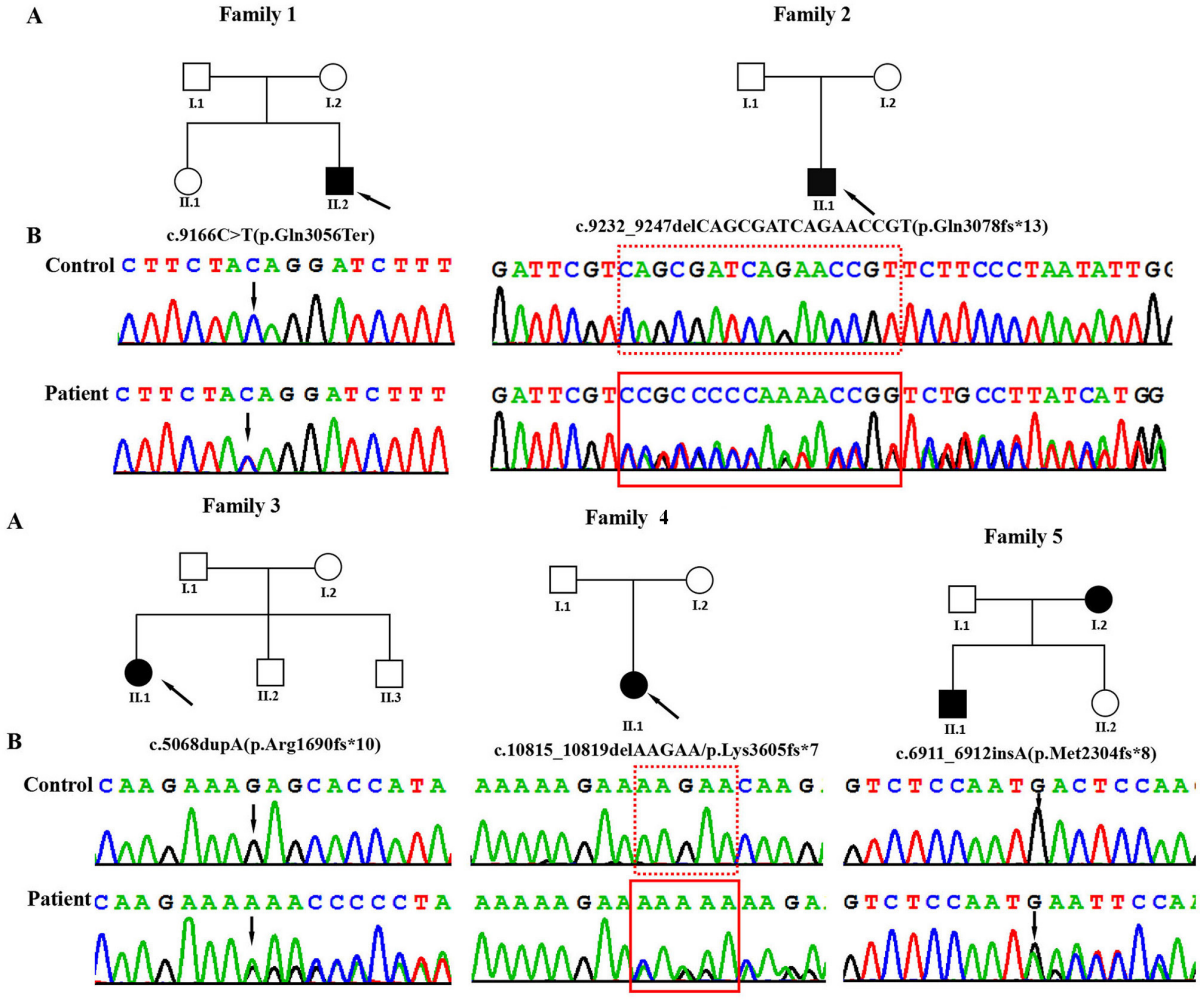
**(A)** Pedigrees of the affected families. Probands are denoted by arrows. **(B)** The Sanger chromatograms of the detected variants in probands of families 1–5. Among them, P1 [NM_170606.3:c.9166C>T (p.Gln3056Ter)] had a non-sense variant, and P2 [c.9232_9247delCAGCGATCAGAACCGT (p.Gln3078fs*13)], P3 [c.5068dupA (p.Arg1690fs*10)], P4 [c.10815_10819delAAGAA/p.Lys3605fs*7], and P5 [c.6911_6912insA (p.Met2304fs*8)] had frameshift variants.

A total of five individuals (four male patients and one female patient) with NDD from unrelated families underwent WES (Figure 1). Genomic DNA was extracted from 5 ml of peripheral blood of the patients and their family members using a Lab-Aid DNA kit (Zeesan Biotech Co., Ltd., Xiamen, China). Whole-exome capture was performed using an Agilent SureSelect V5 enrichment capture kit (Agilent Technologies, Santa Clara, CA, USA), followed by sequencing on the Hiseq2000 platform to generate 100-bp paired-end reads. The sequence reads were aligned to the human genome assembly GRCh37 using the Burrows–Wheeler aligner (BWA-MEM, version 0.7.10). TGEX was utilized to annotate and classify all the variants. The variants were filtered based on specific criteria, including (a) the exclusion of non-rare (MAF ≥ 0.01) variants in the public database (e.g., 1,000 Genomes Project, dbSNP, and Genome Aggregation Database), (b) mutations in the exonic region and splice regions, and (c) predicted deleterious effects by MutationTaster, CADD, SIFT, or PolyPhen2. The pathogenicity of identified variants was interpreted and classified according to the American College of Medical Genetics (ACMG)/Association of Molecular Pathology (AMP) guidelines (19).

## Results

### Clinical features

Patient 1 was a 3-year and 12-month-old boy, the second child of healthy, unrelated Chinese parents. He was born full-term with a birth weight of 3,250 g and a body length of 48.5 cm. At 3 years and 10 months, he was admitted to the Pediatric Intensive Care Unit of the Guangxi Zhuang Autonomous Region Maternal and Child Health Hospital due to hypoxic acute respiratory failure caused by pneumonia. His developmental milestones were globally delayed, with independent sitting at 9 months, standing with support at 20 months, and walking without support at 25 months. He began speaking single words by the age of 2 years and started forming sentences by the age of 3 years. He had moderate ID and behavioral abnormality, including stereotypies such as body rocking, clapping and hand flapping, spinning, hitting objects, and self-talking. Physical examination revealed that the patient was severely underweight (weight: 9 kg, BMI: 10.9 kg/m^2^) and had a short stature (height: 91 cm, −3 SD). Unfortunately, due to economic factors and an unfavorable prognosis, the patient discontinued treatment and passed away a month after discharge.

Patient 2 was a 1-year-old male child, born at full term but small for gestational age, with a birth weight of 2,300 g (< 10th centile), a body length of 44 cm, and a head circumference of 32.4 cm (< 10th centile). He presented with severe ID and global developmental delay. At 11 months, his Full Scale IQ was 43, according to the Wechsler Intelligence Scale for Children. He was unable to stand or walk independently and showed no signs of speech development. Additionally, he experienced postnatal growth retardation with short stature (−2 SD), weight loss (−2.1 SD), and microcephaly (−1.9 SD). The child also exhibited midface hypoplasia and strabismus.

Patient 3 was a female child born at 38 weeks of gestation with normal weight, length, and head circumference. At 13 months of age, she was initially assessed at the Child Healthcare Department of the Guangxi Zhuang Autonomous Region Maternal and Child Health Hospital for feeding difficulty, comprehensive developmental delay, and failure to thrive. She exhibited mild developmental delay, began walking at 15 months, and started talking at 1 year and 8 months. She continued to display short stature (−2.1 SD), mild development delay, speech delays, and learning disabilities. At the age of 8 years, she was readmitted for learning difficulty and hyperactivity and was diagnosed with attention-deficit-hyperactivity disorder (ADHD) and weight loss (BMI: 10.8 kg/m^2^). Treatment with an amino acid-based high-energy formula, high-energy high-protein diet, and aripiprazole (1.25 mg/day) resulted in a 6-kg weight gain after 1 year. However, her ADHD symptoms persisted. At the age of 9 years and 1 month, she began taking 18 mg/day of methylphenidate accompanied by behavioral interventions implemented by her parents, leading to a significant alleviation of her ADHD symptoms. In the most recent examination at the age of 9 years and 5 months, she exhibited mild intellectual disability (Full Scale IQ, 69; Verbal Comprehension Index, 70; Perceptual Reasoning Index, 64; Working Memory Index, 68; and Processing Speed Index, 65). Minor facial dysmorphisms, including prominent eyebrows, a thick lower lip, and a short nose, were observed.

Patient 4 was a 14-year-old boy, the first child of healthy non-consanguineous Chinese parents, who presented with severe short stature (< -4.1 SD). An arginine clonidine GH stimulation test yielded an IGF-1 level of 113 ng/ml (reference range 49–283), an IGF-BP3 level of 2.89 μg/ml (reference range 1.0–4.7), and a peak GH level of 12.65 ng/ml, ruling out the GH deficiency. He demonstrated normal gross and fine motor skills, with a bone age of 12 years. His general performance on the language scale was 80 and below the average range (86%). Expressive language skills were within the normal range, while receptive language skills were impaired with scaled scores of 10 and 5, respectively. The patient also experienced sleep disturbances, including insomnia, difficulty falling asleep, abnormal sleep duration, excessive daytime sleepiness, and disrupted circadian rhythms.

Patient 5, the first child of healthy non-consanguineous Chinese parents, was delivered at 34 weeks plus 2 days of gestation due to premature rupture of membranes. He was 8 years old at the time of the last physical examination. He began walking at 18 months and exhibited hypotonia and talking at 40 months. He had moderate intellectual disability (Wechsler Intelligence Scale for Children-IV, Full Scale IQ = 48). Behaviorally, he exhibited a typical ASD phenotype with speech impairment, social interaction impairment, poor response when called, communication difficulties, behavioral problems, and hyperactivity. He had some dysmorphic features, including a flattened midface, everted lower lip, and esotropia. It is noteworthy that his mother exhibits a mild cognitive impairment, with no other apparent dysmorphic features detected in the family history report.

### Molecular analysis

Using WES, we identified five heterozygous variants in the *KMT2C* gene in patients 1–5 as follows: c.9166C>T (p.Gln3056Ter) in patient 1, c.9232_9247delCAGCGATCAGAACCGT (p.Gln3078fs^*^13) in patient 2, c.5068dupA (p.Arg1690fs^*^10) in patient 3, c.10815_10819delAAGAA(p.Lys3605fs^*^7) in patient 4, and c.6911_6912insA (p.Met2304fs^*^8) in patient 5 (Figure 1). These five variants were validated by Sanger sequencing. Additionally, it was shown that the c.6911_6912insA (p.Met2304fs^*^8) variant was inherited from the mother of patient 5, while the other four variants were not present in the samples of the four parents in the study. Specifically, c.9232_9247delCAGCGATCAGAACCGT (p.Gln3078fs^*^13), c.5068dupA (p.Arg1690fs^*^10), c.10815_10819delAAGAA (p.Lys3605fs^*^7), c.6911_6912insA (p.Met2304fs^*^8), and c.2519_2520dup (p.Thr841fs^*^37) were novel variants, which were not reported in gnomAD, Human Gene Mutation database, 1,000 Genomes Project, Exome Sequencing Project, ExAC, ClinVar, and the Single Nucleotide Polymorphism database. The c.9166C>T (p.GLn3056^*^) non-sense variant is reported in the gnomAD database with a low allele frequency (1 mutated allele out of 833,108 alleles). While this variant has not been previously associated with a disease, multiple computational pieces of evidence predict that it is damaging. These variants were predicted to be deleterious by *in silico* tools. The pathogenicity prediction analysis and ACMG/AMP rating of the five KMT2C variants are shown in Table 2.

**Table 2:** Predicted pathogenicity of novel or *de novo* KMT2C variants.

**Patient**	**Variant (NM_170606.3)**	**Inheritance**	**Affected exon**	**MutationTaster**	**ACMG/AMP**
Patient 1	c.9166C>T (p.Gln3056Ter)	DNM	E38	D	P [(PVS1 + PS2 + PM2_Supporting)]
Patient 2	c.9232_9247delCAGC GATCAGAACCGT (p.Gln3078fs^*^13)	DNM	E38	D	P [(PVS1 + PS2 + PM2_Supporting)]
Patient 3	c.5068dupA (p.Arg1690fs^*^10)	DNM	E36	D	P [(PVS1 + PS2 + PM2_Supporting)]
Patient 4	c.10815_10819delA AGAA (p.Lys3605fs^*^7)	DNM	E34	D	P [(PVS1 + PS2 + PM2_Supporting)]
Patient 5	c.6911_6912insA (p.Met2304fs^*^8)	Maternal	E36	D	P [(PVS1 + PM2_Supporting)]

DNM, *de novo* mutation; D, disease causing; P, pathogenic; LP, likely pathogenic.

## Discussion

*KMT2C*, also known as *MLL3*, is a histone lysine methyltransferase enzyme that plays an important role in modifying histone proteins (6, 8, 20, 21). It is involved in H3K4me1 and H3K4me3 marks related to active enhancers or transcriptionally active regions, respectively (22, 23). *KMT2C* is part of the protein complex associated with Set1 (COMPASS complex), which plays a vital role in eukaryotic developmental signaling pathways (24–26). *KMT2C* knockout mice display ASD-like repetitive behaviors, social deficits, and ID (27). In human, variants in *KMT2C* have been reported to be associated with ASD-like behaviors and Kleefstra syndrome-2 (18, 21, 25, 28–32). Notably, only 13 patients have been reported with variants in *KMT2C*. Among these patients, only one long-term report showed a long-term persistent phenotype. Therefore, the phenotypes associated with *KMT2C* mutations remain incompletely characterized. In this study, we found five *KMT2C* variants related to neurodevelopmental abnormalities. The patients exhibited common disease phenotypes, including mild-to-moderate ID and DD, failure to thrive, facial dysmorphism, speech delay, hypotonia, feeding difficulties, short stature, weight loss, microcephaly, and behavioral abnormalities.

In the patients described in this study, five variants were identified in *KMT2C*, including one non-sense variant and four frameshift variants. *In silico*, these null variants may cause a premature termination codon or a translational frameshift, leading to significantly reduced truncated proteins and markedly reduced mRNA levels due to NMD degradation. These variants were assessed as likely pathogenic or pathogenic according to the ACMG/AMP guidelines (Table 2).

Patient 5 harbors a potentially pathogenic heterozygous variant in the *KMT2C* gene inherited from his mother. Inconsistent phenotypes were observed in patient 5 and his mother. Patient 5 manifested a more extensive and severe phenotype, including delayed motor development, severe language impairment, moderate intellectual disability, autistic behaviors, ADHD, and dysmorphic facial features, while his mother exhibited only a mild cognitive impairment. Consequently, these findings suggest a high degree of variability in the clinical features among individuals with heterozygous mutations in the *KMT2C* gene.

In the current study, we describe the genotype and clinical phenotype of five patients with variants in *KMT2C*, bringing the total number of reported individuals to 18. The list of reported variants of *KMT2C* and the clinical phenotype of our patients and the other reported patients are summarized in Table 3 and Supplementary Table S1 (8, 14–18). Except for one missense variant and one in-frame deletion, all reported variants were loss-of-function variants (LoF; including non-sense variants, splicing variants, frameshifts, in-frame, and microdeletions). Some common phenotypes were observed in patients with these variants. All 18 patients exhibited a range of intellectual disability from mild to severe; 88% of the patients (15/17) displayed diverse minor facial dysmorphisms, including midface hypoplasia, thick and everted lower lip, abnormal eyebrows (arched or prominent), nose abnormalities (saddle bridge, bulbous tip nose, and short nose), and eye abnormalities (deep set eyes, strabismus, ptosis, down-slanting palpebral fissures, and hypertelorism). Speech delay was observed in all patients (17/17), ranging from delayed speech to remaining non-verbal after the age of 15 years. Nearly all patients (14/15) presented with motor delay or dyskinesia. All patients learned to walk, but some did not learn to walk until after the age of 4 years. Behavioral abnormalities were also observed in most patients (13/16) and became more characteristic with age. These abnormalities included autism, ADHD, sleeping disorder, aggressiveness, hyperactivity, automutilation, and elective mutism. Hypotonia was common (11/16), with one patient exhibiting a broad gait and another showing developmental regression. Short stature (12/17) and weight loss (11/17) were the prevalent features, both falling 2–4 standard deviations below the mean for the general population. Aripiprazole medication effectively improved weight in patients with low body weight; two patients, including ours, showed significant weight gain after aripiprazole medication, particularly in abdominal fat. Relative microcephaly was observed in nine out of 18 patients, while macrocephaly was noted in two patients. Additionally, various other variable dysmorphic features, such as brain abnormalities, seizures, phenylketonuria, recurrent respiratory infections, cryptorchidism, bifid uvula, bilateral inguinal hernia, delayed puberty, hearing impairment, insensitivity to pain, constipation, slight telelia, ligamentous hyperlaxity, and eczema, were also observed.

**Table 3:** Clinical features of patients with KMT2C variants.

**Patients clinical data**	**Total**
	***N*** = **18**
Height (SD)	Short statue = 12/17
Weight (SD)	Weight loss = 11/17
OFC (SD)	Microcephaly = 7 • Macrocephaly = 2
Failure to thrive	11/17
Walking	15/16
Speech impairment	17/17
Intellectual disability	18/18
Facial dysmorphism	15/17
Muscular hypertonia	11/16
Behavioral abnormalities	13/16
Brain radiologic features	4/11
Seizures	3/18

Neurodevelopmental disorders caused by chromatin-regulated protein-encoding genes often exhibit overlapping clinical manifestations (33–35). Comparing the clinical manifestations of these different related disorders can enhance our understanding of the broad clinical spectrum of these diseases and the significance of WES in the diagnostic process (36, 37). Although some consistent clinical manifestations of KLEFS2 have been observed, we currently cannot establish a clear correlation between the genotype and the phenotype, which is attributed to the complexity and heterogeneity of the KLEFS2 phenotypes. The diverse phenotype observation indicates that variants occurring at different positions may have site-specific effects on the phenotypes. Moreover, in this cohort of 18 patients, the male-to-female ratio was 1:1. Some severe phenotypes are more likely to be observed in women, including severe ID (4/9), severe speech impairment (3/9), brain abnormalities (3/9), and macrocephaly (2/9), suggesting that sex may influence the outcome. The underrepresentation of patients in this cohort may reflect ascertainment bias, and further confirmation of the observations is expected as the number of patients increases.

The exact mechanisms underlying how these variants cause ID, facial dysmorphisms, language and motor delays, behavioral abnormalities, hypotonia, short stature and weight loss, and additional clinical symptoms remain unclear. The *KMT2C* gene is highly conserved from archaea to eukaryotes and is widely expressed in human tissues with high levels in the cerebellum of the developing and adult human brain (38, 39). This gene encodes an enzyme that monomethylates lysine 4 of histone H3 (H3K4me1) and epigenetically regulates many gene expressions in the brain through chromatin modification (40). In 2017, Koemans et al. found that KMT2C knockdown drosophila exhibited severe defects in memory formation (8). Moreover, they found that KMT2C binds to the promoters of many genes that play important roles in neuronal processes (6). *In vitro* experiments show that reduced KMT2C expression leads to a decrease in the overall level of histone tail modifications H3K4me1 and H3K4me3 (6, 41). Furthermore, rare *KMT2C* variants have been associated with Kleefstra syndrome, ID, ASD, schizophrenia, non-syndromic primary failure of tooth eruption, and a family with colorectal cancer and acute myeloid leukemia (8, 14–18, 42). These findings suggest that the *KMT2C* variant has pleiotropic effects. Functional studies of these variants are needed to fully understand KLEFS2 and its mechanisms of action.

## Conclusion

In conclusion, we present detailed clinical and phenotypic information on five additional unrelated Chinese patients with five novel heterozygous KMT2C variants, expanding the mutational spectrum of KLEFS2. This is the first report of the c.9232_9247delCAGCGATCAGAACCGT (p.Gln3078fs^*^13), c.5068dupA (p.Arg1690fs^*^10), c.10815_10819delAAGAA (p.Lys3605fs^*^7), and c.6911_6912insA (p.Met2304fs^*^8) variants in the *KMT2C* gene. Our study also indicates that there is currently no significant correlation between the genotype and phenotype, providing a foundation for clinical diagnosis and genetic counseling for KLEFS2.

## Data availability statement

The datasets presented in this article are not readily available because of ethical and privacy restrictions. Requests to access the datasets should be directed to the corresponding author.

## Ethics statement

The studies involving humans were approved by the Institutional Review Board and Ethics Committee of Guangxi Maternal and Child Health Hospital. The studies were conducted in accordance with the local legislation and institutional requirements. Written informed consent for participation in this study was provided by the participants' legal guardians/next of kin. Written informed consent was obtained from the individual(s), and minor(s)' legal guardian/next of kin, for the publication of any potentially identifiable images or data included in this article.

## Author contributions

QY: Conceptualization, Data curation, Formal analysis, Funding acquisition, Software, Writing—original draft, Writing—review & editing. QZ: Data curation, Methodology, Validation, Writing—review & editing. SheY: Writing—review & editing. SZ: Writing—review & editing. ShaY: Writing—review & editing. XZ: Writing—review & editing. ZQ: Writing—review & editing. BC: Writing—review & editing. JL: Writing—review & editing.
